# Epidemiology of Injuries during Judo Tournaments

**DOI:** 10.1155/2023/2713614

**Published:** 2023-02-18

**Authors:** Jeroen Mooren, Amber L. von Gerhardt, Irene T. J. Hendriks, Johannes L. Tol, Sander Koëter

**Affiliations:** ^1^Canisius Wilhelmina Hospital (CWZ), Department of Sports Medicine, Nijmegen, Netherlands; ^2^Amsterdam UMC, University of Amsterdam (UvA), Department of Orthopaedic Surgery and Sports Medicine, Amsterdam, Netherlands; ^3^Academic Center for Evidence-Based Sports Medicine (ACES), Amsterdam UMC, Amsterdam Movement Sciences (AMS), Amsterdam, Netherlands; ^4^Amsterdam Collaboration on Health and Safety in Sports (ACHSS), Amsterdam UMC IOC Research Center of Excellence, Amsterdam, Netherlands; ^5^Canisius Wilhelmina Hospital (CWZ), Department of Orthopaedic Surgery, Nijmegen, Netherlands

## Abstract

**Objective:**

To determine the injury incidence proportion, distribution of injuries by anatomical location; injury type; injury severity, time loss; mechanism and situations of injuries; and the relative risk of injuries by gender, age, and weight categories during judo tournaments. *Study Design*. It is a systematic review. *Data Sources*. A systematic review of the literature was conducted via searches in PubMed, EMBASE, Web of Science, CINAHL, SPORTDiscus, Google Scholar, and PEDro. *Eligibility Criteria*. All original studies on the incidence of injuries during judo tournaments were included.

**Results:**

Twenty-five studies were included out of the 1979 studies. Using the modified AXIS tool score for quality assessment, seven were rated as having good quality, nine were rated as having fair quality, and four were rated as having poor quality. The injury incidence proportion during tournaments ranged from 2.5% to 72.5% for injuries requiring medical evaluation and 1.1% to 4.1% for injuries causing time loss (i.e., inability to continue game participation). The most commonly reported injury location was the head, followed by the hand, knee, elbow, and shoulder. The most frequent types of injury were sprains, followed by contusions, skin lacerations, strains, and fractures. In judo tournaments, injuries were more often sustained during standing fights (tachi-waza) than in ground fights (ne-waza).

**Conclusion:**

The tournament injury incidence proportion ranged from 2.5% to 72.5% for injuries requiring medical attention and 1.1% to 4.1% for injuries causing time loss. The head was the most frequently injured body part, and sprain was the most frequent injury type. However, current reports on injuries during judo tournaments are heterogeneous and inconsistent, limiting our understanding of in-match injury risks. Future studies should utilize the guidelines of the International Olympic Committee consensus meeting statement on the methodological approach to injury reporting. We recommend a judo-specific extension of this statement to fit the unique features of judo sports practice.

## 1. Introduction

Judo is a worldwide popular sport with more than 20 million practitioners in 200 countries [1]. It is a full-contact sport with a relatively high incidence of injury during tournament participation. The reported tournament injury incidence proportion is highly variable and ranges from 11.2 to 29% [2]. The reported injury incidence rate per 1000 athlete exposures (in fights) ranges from 41.2 to 81.6 [2]. Its competition rules have changed several times in the past two decades [3, 4]. Some of these rule changes were specifically meant to reduce injuries. However, the effect of these rule changes on the injury incidence is unknown. Epidemiological data on judo injuries in tournaments can guide injury prevention programs and help researchers evaluate the effect of rule changes on the injury incidence.

Two previous systematic reviews describe the epidemiology of judo injuries. Pocecco et al. [2] published a comprehensive summary of judo injuries in competition and training in 2013. The most common injury type was a sprain, followed by strain and concussion. The most affected body locations were the knee, shoulder, and hand/fingers. Bromley et al. [5] published a systematic review on injury in Olympic combat sports (judo, taekwondo, wrestling, and boxing) in 2017. They included prospective studies of injury and illness lasting more than 12 weeks sustained in training and competition. For judo, they found an injury incidence rate of 4.2 per 1000 hours. The body locations with the highest incidence proportion of injury were the lower back, shoulder, and knee. Men had higher injury incidence rates than women.

However, the conclusions of Pocecco et al. must be re-evaluated because, since 2013, new studies have been published [6–21]. In the study by Bromley et al., only two studies met the inclusion criteria for judo [22, 23]. Both the included studies had a very small sample size, which limits researchers' ability to draw conclusions and increases the risk of over or underestimating incidence rates [22, 23]. Furthermore, the injuries of elite judokas cannot be generalized to the general population.

Therefore, to conclude, there is currently no systematic review that describes the currently existing literature on injuries during judo tournaments. We decided to focus on injuries during judo tournaments specifically because the injury incidence and injury characteristics in judo training cannot be extrapolated to competition, and the injury incidence proportion during judo tournaments is high, as with most combat sports [24, 25]. Therefore there is a need for preventive strategies. The burden of sport-specific injuries can guide future injury prevention programs and can help researchers evaluate the effect of rule changes on injury occurrence. Moreover, it is useful for organisers and medical personnel of judo tournaments to have information regarding the injury incidence and the location and type of injuries during judo tournaments. With this epidemiological information, they can prepare themselves properly and arrange sufficient medical personnel and material for the tournament. The aim of this study was to systematically review the injury incidence proportion, distribution of injuries by anatomical location, injury type, injury severity, and time loss, mechanism and situations of injuries, and the relative risk of injuries by gender, age, and weight categories during judo tournaments.

## 2. Methods

This systematic review was performed by following the Preferred Reporting Items for Systematic Review and Meta-Analysis (PRISMA) protocols [26] to ensure appropriate reporting. The protocol is registered in the international prospective register of systematic reviews (PROSPERO) to provide transparency (ID number 181147) [27].

### 2.1. Outcome Measures

The primary outcome was the injury incidence proportion during judo tournaments. Secondary outcome measures were the distribution of injuries by anatomical location; injury type; injury severity, time loss; mechanism and situations of injuries; and the relative risk of injuries by gender, age, and weight categories.

### 2.2. Injury Definition

The injury definitions were categorised, according to the categorisation of Clarsen and Bahr [28], into [1] injuries for which medical assistance was sought, [2] injuries leading to time loss, and [3] other injury definitions or not specified. The first injury definition category was “any musculoskeletal complaint newly incurred due to competition during the tournament that received medical attention regardless of the consequences with respect to absence from competition or training” or variations of this definition. The second injury definition was “all injuries that resulted in an interruption of practice (competition or training).” Other injury definitions, which did not fit in “requiring medical attention” or “leading to time loss,” are mentioned in the injury definition table. Injuries are described in terms of the injury incidence proportion, a proportion of the injured athletes to the total number of athletes per tournament (%), or injury incidence rates per 1000 athlete exposures.

### 2.3. Eligibility Criteria

All original studies that were published in a peer-reviewed journal and described the incidence of injuries during judo tournaments were considered eligible for inclusion. Exclusion criteria were as follows: articles written in languages other than English, German, Dutch, or French. Reviews, case report studies, and anecdotal reports were excluded. Furthermore, articles about Paralympic or visually impaired judo were excluded. Studies that used the injury data from the same tournament as another study were excluded. Studies that only reported data about one specific injury type were also excluded.

### 2.4. Search Strategy

We searched for terms on “judo” and “injuries” or synonyms for injuries (full-search strings are available in Appendix). The following databases were selected for the search: PubMed, EMBASE, Web of Science, CINAHL, SPORTDiscus, Google Scholar, and PEDro. For Google Scholar, we listed the results by relevance and used the first 200 results for analysis. This method was described by Bramer et al. [29] in their study on the optimal database combination for systematic literature reviews. The reference lists of all included publications and relevant systematic reviews were checked, and forward citation searches were performed.

### 2.5. Study Selection

**Figure 1:**
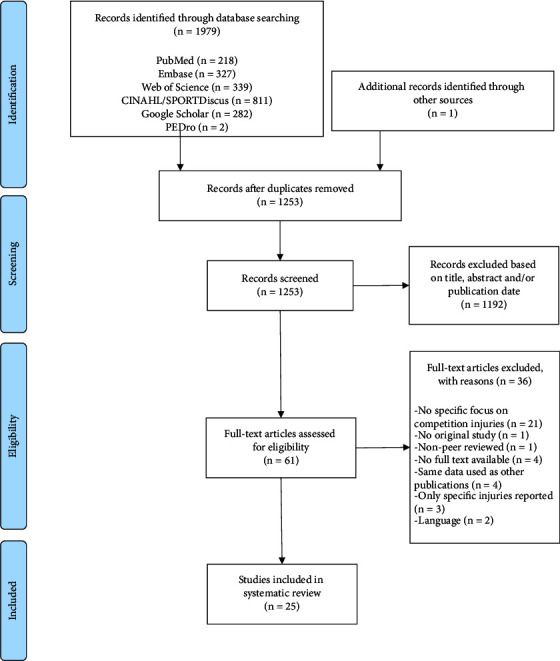
PRISMA flow diagram of the study selection.

After the search was completed, all duplicates were removed. Two authors (JM and AvG) independently screened all titles and abstracts found in the search and assessed them for eligibility, using the web application, Rayyan QCRI. [30] After the first selection was made, full-text articles from all articles were retrieved. The aforementioned authors then read all the articles and made the final selection. The selection process is further described in Figure 1. In case of disagreement, a consensus was sought through discussion among the two authors.

### 2.6. Data Extraction

All studies were listed in a standardised data extraction form, as adapted from the Cochrane Collaboration. In this spreadsheet, we listed whether each study mentioned the following injury characteristics: injury incidence, definition, location, type, mechanism and situation, severity, time loss, and distribution of injuries across age, gender, and weight classes. A new spreadsheet was made for each of these characteristics, in which the data concerning this injury characteristic were listed. Because there were no uniform categorisations used in different studies for injury types and injury locations, we recategorised these data according to the IOC consensus statement by Bahr et al. [31] for injury locations and [6] injury types. The study authors were contacted in case of missing information.

### 2.7. Quality Assessment

We modified the Critical Appraisal Tool for Cross-Sectional Studies (AXIS tool) [32] specifically for the quality assessment of judo injury epidemiology studies (Supplementary Appendix A). We removed questions about sampling, as these questions were not applicable to the included studies, and added three different questions on exposure (number of matches), injury definitions, and outcomes in terms of injury incidence and injury characteristics. Two authors (JM and AvG) independently assessed the quality of the included articles with this modified version of the AXIS tool. The maximum score is 23.5. The final scores were determined in a consensus meeting. If no consensus was reached, a third author (IH) made the final decision. The definition of a good, fair, and poor quality article was adjusted for the modified version of the AXIS tool. A score equal to or greater than 18 (76.6% of the maximum score) was considered to be of good quality, a score between 12 and 18 was considered to be of fair quality, and a score less than 12 (51.1%) was considered to be of poor quality.

### 2.8. Synthesis of Results

We used descriptive statistics and used tables and figures to show data regarding injury incidence; injury locations; injury types; injury severity; time loss; injury mechanisms and situations; and distribution of injuries across gender, age, and weight categories. For injury types and locations, we listed the three most common types and locations of each study in a figure. We did not perform a quantitative data synthesis (meta-analysis) because the included studies had different study designs, injury definitions (i.e., outcome measure), and populations and were therefore too heterogeneous with regard to group size, population (level of competition), and effect size that we deemed it likely this could cause both clinical and statistical heterogeneity [33].

For differences in the injury incidence between age, gender, and weight classes, which were mentioned in the included studies, we considered a *p* value less than 0.05 to be statistically significant.

## 3. Results

### 3.1. Study Selection and Study Characteristics

The search was performed on 14 November 2022. Figure 1 shows the flow diagram of the selection process of the search. Twenty-five studies were included in this systematic review.  Table 1 provides a summary with descriptive study characteristics, with a total of 361,581 athletes (ranging from 83 to 316,203 per study).

### 3.2. Injury Definitions

The injury definitions that were used in the included studies are listed in Table 2. The most frequent injury definition (*n* = 16) was “a physical complaint, for which assistance was sought from a tournament healthcare professional,” or variations of this definition [6, 7, 10–13, 15, 19, 21, 34–40].

### 3.3. Critical Appraisal

Twenty-five articles were included, of which nine were rated as having good quality (score > 18), ten were rated as having fair quality (score 12–18), and six were rated as having poor quality (score < 12). The mean quality rating was 15 ± SD of 4.4 (64% of the maximum score) and ranged between 4 (17%) and 20.25 (86%). The results of the critical appraisal are presented in Table 3 and in a colour-coded table with the risk of bias assessments per question in Supplementary Appendix B.

### 3.4. Injury Incidence

Twenty-one of the twenty-five included studies described the injury incidence proportion with a range from 1.1% to 72.5% in all three injury definition subgroups (Table 4). First, the incidence proportion for “injuries requiring medical attention” ranged between 2.5% and 72.5%. Second, for injuries leading to time loss, the tournament incidence proportion ranged between 1.1% (>7 days of time loss) and 4.1% (not completing a contest in a tournament). Third, studies with other injury definitions reported an injury incidence proportion between 2.6% and 28.9%. Fifteen studies reported the injury incidence rate per 1000 athlete exposures (IIRAEs) or the total number of fights, by which we could calculate the IIRAE. The reported IIRAE ranges from 10.9 to 109 for injuries requiring medical attention and 4.2 to 60 for injuries causing time loss.

The studies by Lystad et al. and Cierna et al. were the only two studies that reported the injury incidence per 1000 minutes. Cierna et al. found a total of 10.9 injuries per 1000 minutes of exposure. The injury incidence during the Olympic Games found by Lystad et al. was 9.6 per 1000 minutes, i.e., 576 per 1000 hours.

### 3.5. Injuries Requiring In-Hospital Evaluation

Six studies described the number of injuries that required further evaluation in a hospital. The number of injuries that required evaluation in a hospital ranged from 1.5 (Ikumi et al.) [13]to 12.8 (Chirazi and Babiuc) [17] per 1000 athlete exposures. Rousseau et al. [8] and Dah and Djessou [39] described 2.3 and 2.6 hospital treatments per 1000 athlete exposures, respectively. Blach and Smolders [20] described that 0.48% of all competitors needed transportation to a hospital, and they did not describe hospital transportations per 1000 athlete exposures.

### 3.6. Injury Location

**Figure 2:**
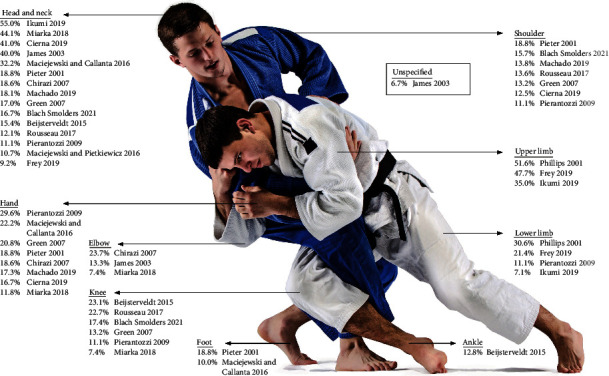
Most common injury locations based on the top 3 of each study (shown in percentage of the total number of injuries per study).

Eighteen studies described the distribution of injuries across body locations. The three most common injury locations for each study are listed and shown in Figure 2. The studies by Frey et al. [14], Maciejewski and Pietkiewicz [10], Ikumi et al. [13], and Phillips et al. [37] did not further specify the number of upper and lower limb injuries. The study by Dah and Djessou [39] showed incomplete and conflicting results for injury locations. Most studies mentioned that head and neck injuries were the most common (*N* = 7) or second most common (*N* = 4). Other locations that were frequently injured were the hand, knee, elbow, and shoulder.

### 3.7. Injury Type

**Figure 3:**
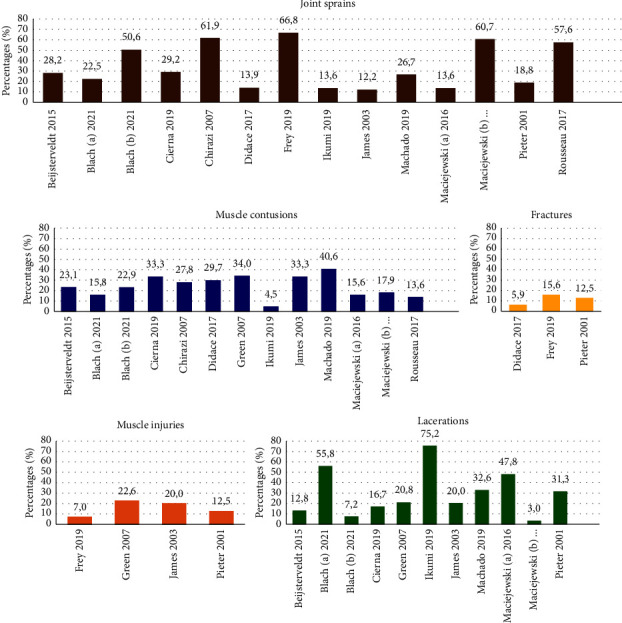
Most common injury types based on the top 3 of each study (shown in percentage of the total number of injuries per study). Corresponding articles: Blach (a) = Blach and Malliaropoulos, Blach (b) = Blach and Smolders, Maciejewski (a) = Maciejewski and Callanta, and Maciejewski (b) = Maciejewski and Pietkiewicz.

Nineteen studies described the distribution of injuries across injury types. The three most common types of injuries for each study are mentioned in Figure 3. The studies by Miarka et al. [9], Dah and Djessou [39], Phillips et al. [37], and Pierantozzi and Muroni [41] used an injury location classification that could not be modified according to the classification used by Bahr et al. [31]. A joint sprain was the most common injury overall. Other frequent injury types, in the order of decreasing frequency, were muscle contusions, lacerations, muscle injuries, and fractures. The study by Frey et al. [14], which only reported injuries leading to time loss, reported the highest relative incidence of joint sprains (66.8%) and fractures (15.6%).

### 3.8. Injury Mechanisms and Situations

Six articles described the mechanisms and situations of injuries in judo tournaments (Table 5). All articles reported that most of the injuries occurred during standing fight (tachi-waza) situations (ranging between 50.0% and 84.9%) compared to ground fights (ne-waza) (ranging between 0.0% and 33.3%). Four articles mentioned a third situation category for prohibited actions or when the situation was not clear. The incidence proportion for this category, “other,” ranged from 3.8% to 28%.

The most common injury mechanism during standing fights is when the judo athlete is being thrown (ranging between 6.7% and 37.0%, the most common mechanism in three studies). Subsequently, performing a throw (ranging between 6.7% and 34%) and grip fights (kumi-kata) (ranging between 6.3% and 32.4%, both common mechanisms in two studies) cause most injuries. The most frequent mechanisms in ground fights where injuries occurred are armlocks (ranging between 3.7% and 13.3%). Choking injuries occurred in up to 8.8% of the mechanisms, possibly resulting in loss of consciousness. Injuries caused by prohibited actions occurred in up to 7.0%.

### 3.9. Distribution of Injuries across Genders

Twelve articles described the differences between the injuries sustained by men and those sustained by women in judo tournaments (Supplementary Appendix C). The influence of gender on the injury incidence proportion during judo tournaments is inconsistent. On the one hand, six articles concluded that men were more prone to injuries than women (the incidence of injuries among men ranged between 1.04% and 14.3%). On the other hand, six articles registered more injuries during judo tournaments among women (the incidence of injuries among women ranged between 1.33% and 12.0%).

### 3.10. Distribution of Injuries across Age Categories

Four articles presented a clear distinction between youth (less than 18 years old) and adult (18 years old and older) athletes with regard to the distribution of injuries (Supplementary Appendix D). Adult athletes (the incidence ranged between 1.3% and 21.0%) sustained more injuries than youth athletes (the incidence ranged between 0.9% and 14.4%) during judo tournaments, according to three out of four articles. However, this difference did not reach statistical significance (Maciejewski and Callanta [11]), or the statistical significance was not mentioned in the article.

### 3.11. Distribution of Injuries across Weight Categories

Six articles reported the distribution of injuries across the three main subgroups of weight categories, namely, lightweights, middleweights, and heavyweights. However, six studies did not use the same categorisation for these weight categories. Therefore, the results are inconsistent (Supplementary Appendix E).

## 4. Discussion

This systematic review focusses on the epidemiology of injuries in judo tournaments. We included twenty-five articles, of which nine were rated as having good quality, ten were rated as having fair quality, and six were rated as having poor quality. The tournament injury incidence proportions ranged from 6.8% [35] to 72.5% [39] for injuries requiring medical attention and 1.1% [14] to 4.1% [42] for injuries leading to time loss. The most commonly injured location overall was the head, followed by the hand, knee, elbow, and shoulder. A sprain was the most common injury type, followed by contusions, lacerations, strains, and fractures.

### 4.1. Injury Definition

The most frequent (*n* = 16) injury definition used was “a physical complaint, for which assistance was sought from a tournament healthcare professional,” or variations of this definition [6, 7, 10–13, 15, 20, 21, 34–40]. This definition is very practicable in its use and provides insight into the workload of health care professionals who encounter at a judo tournament. An inherent limitation of this definition is that it does not take injury severity into account. This is important because many minor injuries that meet this definition cause limited impairment and have no or little effect on athlete performance. A common example is a finger laceration for which a bandage is applied. The use of this definition might overestimate the incidence of injuries irrelevant to the participant, which raises the question whether the use of time loss as criterium for injury is more appropriate in judo. Both definitions underestimate relevant overuse injuries since most athletes continue to participate despite medical problems. None of the studies defined injury irrespective of the need for medical attention or time loss nor used an athlete's perspective on injuries. We suggest using a modified version of the Oslo Sports Trauma Research Centre Questionnaire [43] for all participants in future studies, to take injury severity into account and make sure that injuries without medical attention are registered as well.

### 4.2. Injury Incidence

The large variation in injury definitions explains a great proportion of the wide variety in reported injury incidences. For injuries requiring medical attention, the reported injury incidence proportion varied between 2.5% [35] and 72.5% [11] for all competitors. For injuries leading to time loss, the reported incidence proportion ranged between 1.1% [14] and 4.1% [42] for total competitors, with four out of five studies reporting an injury incidence proportion between 1.1% [14] and 2.6% [8, 36].

In the past two decades, judo competition rules have changed several times. These rule changes consisted of a change in duration of the contest, scoring system, golden score, and gripping rules [3]. Furthermore, some specific actions were prohibited in order to reduce injuries. The current competition rules are designed to ensure that judo causes as little trauma as possible [4]. In our review, we did not evaluate the effect of rule changes on the injury incidence. The variation in the methodological approach, injury definition, and relatively small sample size of some of the included studies will make it difficult to evaluate this effect with the current literature.

The incidence rates of injury in judo are higher than the reported injury incidence rates in Brazilian jiu-jitsu (BJJ). Scoggin et al. [44] reported an injury incidence rate of 9.2/1000 exposures requiring medical attention in eight statewide BJJ tournaments in Hawaii. In our review, not all studies reported the injury incidence rate per 1000 athlete exposures (IIRAEs). The studies that did so reported an IIRAE between 22.7 [13] and 115 [39] for injuries requiring medical attention. The difference in BJJ may be caused by the fact that BJJ matches take place on the ground (ne-waza) for a larger part of the contest than in judo, while most injuries occur during standing fights (tachi-waza) [9, 10, 36, 38, 41, 42]. The injury incidence rate in judo is lower than in mixed martial arts (MMAs). In a recent systematic review, Lystad et al. [45] described an IIRAE between 110.4 and 473.5 per 1000 in MMA in their systematic review, but reporting of injury definitions in the included studies was inconsistent. The difference in the injury incidence is likely due to the fact that, in MMA, striking and kicking is allowed, and MMA matches consist of multiple rounds and therefore a longer duration of the athlete's exposure.

In another systematic review by Lystad et al., the injury incidence during three consecutive Olympic Games (2008–2016) in judo was compared with the incidence in the other olympic combat sports, taekwondo, boxing, and wrestling, respectively [21]. The observed IIRAEs, in descending order, were 76.6 in boxing, 46.4 in taekwondo, 34.0 in judo, and 22.7 in wrestling, respectively. In this study, the injury incidence rates in sports that involve kicking and striking are also higher than those in judo.

### 4.3. Injury Location

The most frequently injured body location in the included studies was the head. This is different [3, 4] from the results of Pocecco et al., who reported the knee, shoulder, and hand/fingers to be most commonly injured. For injuries leading to time loss, however, the head was not the most affected injury location in our review. In the study by Frey et al. [14], in which only injuries leading to time loss were reported, 3511 injuries occurred in 316203 competitors. Only 54 of these injuries were head traumas with loss of consciousness, another 54 were fractures of the face, and 47 cases were of loss of consciousness resulting from a choke. These injury incidence proportions of the head are much smaller than those in other studies, suggesting that a large proportion of the reported head injuries in other studies consist of minor traumas such as lacerations and contusions that do not result in time loss. This is applicable to hand injuries as well. Moreover, although hand injuries were common in the included studies that used medical attention as part of the injury definition, the hand injury incidence proportion was less than 4% in an online survey for severe injuries with more than three weeks of time loss in 4659 judokas according to Akoto et al. [46].

In our systematic review, the knee and shoulder were also frequently injured body locations. This is consistent with the findings of Akoto et al. [46], who found the knee and shoulder to be the most affected body parts (both 23% of all severe injuries), and this is also in line with the study by Kim et al. [23], in which they reported on a four-year prospective injury surveillance study at the training centre in South Korea for national-level athletes and found knees and shoulders to be the second and third most frequently injured body locations. In contrast to our systematic review, they found the lower back to be the most frequently injured body part (10.9%). This difference may be caused by the fact that judokas might not seek medical attention for overuse injuries and injuries with a gradual onset during a tournament.

The elbows were among the top three most commonly injured body parts in three of the included studies [9, 17, 36]. These injuries were relatively common in the studies by Akoto et al. [46](reported by >4% of all athletes) and Kim et al. [23] (7.5% of all injuries) as well.

### 4.4. Injury Type

A sprain was mentioned as the most frequent injury type in most studies. For injuries causing time loss, a sprain was reported to be the most frequent injury type in all studies except for the study by Pieter et al. [38]. Frey et al. [14] reported sprains in 66.8% of all injuries, James and Pieter [36] in 100%, Pieter et al. [38] in 33.3%, and Rousseau et al. [8] in 57.6%. For lacerations and contusions, which are the second and third most common injury types overall, frequencies are higher for injuries requiring medical attention than those for injuries leading to time loss. All the three studies that compared injuries between male and female athletes found more knee sprains (including anterior cruciate ligament injuries and medial collateral ligament injuries) in female athletes [14, 36, 38]. This may be due to anatomical differences, hormonal differences, and differences in joint laxity. Frey et al. [14] reported more elbow dislocations in female athletes, while male athletes were more prone to shoulder dislocations. Miarka et al. [9] proposed that these sex differences might be related to the fighting style, with male judokas being more aggressive, having more full-body contact with their opponents, and having more contact with the mat. This is consistent with the finding of Didace et al. [16], who reported more contusions and distortions in woman and more fractures in men.

### 4.5. Injury Mechanisms and Situations

Six articles described the mechanisms and situations of injuries in judo tournaments [9, 10, 36, 38, 41, 42]. All articles reported that most injuries occurred during standing fight (tachi-waza) situations compared to ground fight ones (ne-waza). This is likely caused by the fact that, considerably, more time is spent in tachi-waza than in ne-waza [47]. Furthermore, throws (performing a throw and being thrown) carry a relatively high injury risk given the force and speed they require, as well as the impact of the mat, respectively. Two studies [13, 38] reported that, during grip fights (kumi-kata), male athletes are exposed to more hand and finger injuries requiring medical attention. Contrarily, female athletes spend relatively more time in ground fights [47] causing elbow dislocations or medical collateral ligament elbow injuries caused by armlocks [14]. In standing fights, more upper limb injuries were reported in female athletes than in male athletes after being thrown [42].

### 4.6. Strengths and Limitations

The main strength of this systematic review is that we used an extensive search strategy with broad search terms in five different electronic databases. Database searching was supplemented by extensive hand-searching of all references of the included studies. We performed a quality assessment of the available literature and took the injury definition into account when analysing the study results.

Limitations of the literature include lack of uniformity in injury definitions and classification of injury types, locations, severity, mechanisms, and weight categories. Because of the heterogeneity of the total group (with different injury definitions, populations, level of competitions, group sizes, and exact outcome measures), we considered a formal meta-analysis of limited additional values. We were able to calculate the percentage of injured competitors per tournament but were unable to calculate the number of injuries per 1000 athlete exposures for every study due to missing data. The latter is a more efficacious way of defining the injury incidence, as the number of fights per competitor and tournament can vary widely. The injury incidence defined as injuries relative to time will be even more efficacious as we are aware that not all athlete exposures are equal. Nevertheless, this time-related injury definition was not feasible for the current available literature.

The quality of the scientific methods used in different studies varied. We measured quality by using a new critical appraisal tool based on the AXIS tool [32]. This is an objective way to rate studies and reduce the risk of nonsystemic bias.

A possible publication bias is that the included studies were mostly based on high-level tournaments. Therefore, the current data might not be representative for tournaments on a lower level.

### 4.7. Future Directions

A uniform, reliable, and valid methodological approach can contribute to better injury surveillance studies in judo. This can help researchers develop better injury prevention protocols to mitigate preventable injuries and evaluate the effect of rule changes on the injury incidence. In 2020, the International Olympic Committee published a consensus statement regarding the methodology for injury surveillance studies [31]. Based on the IOC consensus statement, authors anticipated that sport-specific statements would provide further recommendations; this has been recently performed by Verhagen et al. for tennis [48]. The IOC consensus statement cannot be generalized to judo because judo has variable lengths of contests, a variable number of contests per tournament, specific mechanisms, throws and situations in which injuries occur, and specific individual variables such as the weight class, competition level, and grading system for the belts that judokas wear. In addition, there are some judo-specific injuries, such as loss of consciousness by strangulation, which do not conform to the injury-type categories proposed in the IOC statement. Therefore, we recommend a judo-specific extension of this IOC statement. This judo-specific consensus statement should contain the following methodological topics: injury definitions, data collection methods, athlete exposure and study population characteristics, a classification of injury types, severity, body locations, and injury mechanisms and situations. We propose a broad injury definition in order to register any physical complaint from or during a tournament, irrespective of time loss or medical attention. Injuries should then be further classified as injuries with or without time loss. Data analysis could be performed more easily by video analysis.

## 5. Conclusion

The tournament injury incidence rate per 1000 athlete exposures ranged from 10.9 to 115 for injuries requiring medical attention and 4.2 to 60 for injuries causing time loss. The tournament injury incidence proportion ranged from 2.5% to 72.5% for injuries requiring medical attention and 1.1% to 4.1% for injuries causing time loss. The head was the most frequently injured body part requiring medical attention, and a sprain was the most frequent injury type. There was a heterogeneous methodological approach and inconsistent reporting of data in various included studies. We advise future studies to follow the guidelines of the IOC consensus meeting statement on the methodological approach to injury reporting [31]. Furthermore, a judo-specific extension of this statement is suggested in order to achieve an optimal uniformity in the methodological approach and make the results useful for injury prevention programs.

## Figures and Tables

**Table 1:** Descriptive study characteristics.

Author	Year	Country	Study design	Data collection		Competitors	Gender (M/F)	Age
Beijsterveldt et al.	2015	Netherlands	Prospective	Injury surveillance system		311	Not provided	Youth olympic 15–17 years
Blach and Malliaropoulos	2021	Multinational	Prospective	Injury report by mat physicians		7268	4054/3214	All judoka
Blach and Smolders	2021	Multinational	Prospective	EJU injury registration form		26862	15571/11291	19–35 years
Chirazi et al.	2017	Romania	Prospective	Observations		222	222/0	U23/senior
Cierna et al.	2019	Slovakia	Prospective	Injury report form		295	176/119	U23
Dah et al.	1989	Ivory Coast	Prospective	Injury report form		125	Not provided	Not provided
Didace et al.	2017	Congo	Retrospective	Doctor consult		101	68/33	25.2 ± 1.7 years
Engebretsen et al.	2013	UK	Prospective	Standardised form by head physicians		383	230/153	Olympic
Frey et al.	2019	France	Prospective	Injury surveillance system		316203	237632/78571	All judoka
Green et al.	2007	UK	Prospective	Questionnaire		392	284/108	20.9 years (18–43 years)
Ikumi et al.	2019	Japan	Prospective	Injury report form		Not provided^*∗*^	420	All judoka
James et al.	2003	UK	Prospective	Injury data form		116	70/46	Elite senior
Junge et al.	2009	China	Prospective	Standardised injury report form		385	Not provided	Olympic senior
Laskowski et al.	1995	United States of America	Prospective	Injury report form		11	Not provided	Not provided
Lystad et al.	2020	Multinational	Prospective	IOC injury surveillance system		925	Not provided	Olympic
Machado et al.	2019	Brazil	Retrospective	Injury report form		2890	Not provided	All judoka
Maciejewski and callanta	2016	Philippines	Prospective	Injury form		192	128/64	Junior
Maciejewski and Pietkiewicz	2016	Philippines	Prospective	Injury survey		302	169/133	All judoka
Miarka et al.	2018	Brazil	Prospective, video analysis	Video analysis by ringside doctors		673	415/258	Elite senior
Park et al.	2021	Korea	Prospective	Online questionnaire		317	317/0	All judoka
Phillips et al.	2001	South Africa	Prospective	Assessment by medical staff		210	121/89	Not provided
Pierantozzi et al.	2009	Italy	Retrospective, video analysis	Video analysis		83	51/32	High-level judoka
Pieter et al.	2001	Philippines	Retrospective	Questionnaire	184		100/84	Not provided
Rousseau et al.	2017	France	Prospective, video analysis	Observation form	2458		1268/1189	25.3 years (17–35 years)
Soligard et al.	2017	Brazil	Prospective	Injury surveillance system	673		Not provided	Olympic senior

^*∗*^Number of competitors is not provided; solely, 18470 athlete exposures (AEs) are provided.

**Table 2:** Injury definitions used in each study.

Author	Year	Injury definition category	Injury definition	Data collection
Beijsterveldt et al.	2015	1	Medical attention	Injury surveillance system
Blach and Malliaropoulos	2021	3	Other	Injury report form
Blach and Smolders	2021	1	Medical attention	Injury registration form
Chirazi et al.	2017	3	Other	Observations
Cierna et al.	2019	1	Medical attention	Injury report form
Dah et al.	1989	1	Medical attention	Injury report form
Didace et al.	2017	3	Other: any physical complaint sustained by a competitor irrespective of the need for medical attention or time loss from activities, or which caused an exclusion from sports-related activities for at least 4 days	Doctor consult
Engebretsen et al.	2013	1	Medical attention	Standardised form by head physicians
Frey et al.	2019	2	Time loss: all relevant traumatic injuries that resulted in an interruption of practice (either in competition or training) for more than 1 week	Injury surveillance system
Green et al.	2007	3	Other: a situation in which the judoka either requested medical treatment or was unable to continue a contest	Questionnaire
Ikumi et al.	2019	1	Medical attention	Injury report form
James et al.	2003	1	Medical attention	Injury data form
Junge et al.	2009	1	Medical attention	Standardised injury report form
Laskowski	1995	1	Medical attention	Injury report form
Lystad	2020	1	Medical attention	Injury surveillance system
Machado et al	2019	1	Medical attention	Injury report form
Maciejewski and Callanta	2016	1	Medical attention	Injury form
Maciejewski and Pietkiewicz	2016	1	Medical attention	Injury survey
Miarka et al.	2018	3	Other: all injuries were managed by attending ringside doctors during the competition. Because the recording of injuries was at the discretion of attending ringside doctors, no strict operational injury definition was imposed in this study	Video analysis by ringside doctors
Park et al.	2021	2	Time loss: the failure of participation in competitions or trainings for at least 24 hours within 12 months	Online questionnaire
Phillips et al.	2001	1	Medical attention	Assessment by medical staff
Pierantozzi et al.	2009	3	Other: any physical complaint, which is caused by transfer of energy that exceeds the body's ability to maintain structural and/or functional integrity, which is sustained by a player during a match, irrespective of the need for medical attention or time loss from activities	Video analysis
Pieter et al.	2001	1	Medical attention	Questionnaire
Rousseau et al.	2017	3	Other: all traumas	Observation form and video analysis
Soligard et al.	2017	1	Medical attention	Injury surveillance system

**Table 3:** Modified AXIS scores and allocated quality rating for the included articles.

Author	Year	Modified AXIS	Percentage of the maximum score (%)	Quality rating
Beijsterveldt et al.	2015	19.25	81.9	Good
Blach and Malliaropoulos	2021	14	59.6	Fair
Blach and Smolders	2021	14.5	61.7	Fair
Chirazi et al.	2017	4	17.0	Poor
Cierna et al.	2019	19	80.9	Good
Dah et al.	1989	6	25.5	Poor
Didace et al.	2017	5.5	23.4	Poor
Engebretsen et al.	2013	20.25	86.2	Good
Frey et al.	2019	15.25	64.9	Fair
Green et al.	2007	18.5	78.7	Good
Ikumi et al.	2019	18.5	78.7	Good
James et al.	2003	16.25	69.1	Fair
Junge et al.	2009	15.5	66.0	Fair
Laskowski	1995	12.5	53.2	Fair
Lystad	2020	20	85.1	Good
Machado et al.	2019	14.75	62.8	Fair
Maciejewski and Callanta	2016	18.25	77.7	Good
Maciejewski and Pietkiewicz	2016	19.25	81.9	Good
Miarka et al.	2018	14.5	61.7	Fair
Park et al.	2021	11	46.8	Poor
Phillips et al.	2001	10.25	43.6	Poor
Pierantozzi et al.	2009	8.75	37.2	Poor
Pieter et al.	2001	16.5	70.2	Fair
Rousseau et al.	2017	16.25	69.1	Fair
Soligard et al.	2017	19	80.9	Good

**Table 4:** Injury incidence rates and proportion and incidence proportion of injuries leading to time‐loss.

Author	Year	Injury rate per 1000 athlete-exposures	Injury incidence proportion (%)	Incidence proportion (%) of injuries leading to time-loss
Beijsterveldt et al.	2015		12.5	
Blach et al.	2021		5.5	
Blach et al.	2021		2.5	
Chirazi et al.	2017		8.1	
Cierna et al.	2017	35.6	8.1	
Dah et al.	1989	115	72.5	
Engebretsen et al.	2013		6.8	
Frey et al.	2019	4.2	1.1	1.1
Green et al.	2007	41.3 (male) 40.9 (female)	13.5	4.1^*∗*^
Ikumi et al.	2019	22.7		
James et al.	2003	42.6	12.9	2.6
Junge et al.	2009		9.9	
Laskowski	1995		9.1	
Lystad	2020	34.0		
Machado et al.	2019		16.8	
Maciejewski and Callanta	2016	98.3	46.9	
Maciejewski and Pietkiewicz	2016	38.5	9.3	
Miarka et al.	2018	47.2	10.1	
Park et al.	2021	60		
Phillips et al.	2001	100.3	20	
Pierantozzi et al.	2009	109	28.9^*∗∗*^	
Pieter et al.	2001	32.2	8.7	1.6
Rousseau et al.	2017	10.9	2.7	2.6
Soligard et al.	2017		8.2	

^*∗*^12 competitors were lost to follow-up. ^*∗∗*^The authors analysed only 124 of the fights during 4 international tournaments.

**Table 5:** Distribution (in percentages) of the mechanisms and situations of injuries.

Mechanisms and situations	Study					
	Green et al. [42] (%)	James and Pieter [36] (%)	Maciejewski and Pietkiewicz [10] (%)	Miarka et al. [9] (%)	Pierantozzi and Muroni [41] (%)	Pieter et al. [38] (%)
Tachi-waza	84.9	53.3	73.0	73.5	85.2	50.0
Grip fight	22.6		25.0	32.4	29.7	6.3
Performing throw	24.5	20.0	34.0	11.8	11.1	18.8
Being thrown	28.3	20.0	14.0	29.4	37.0	25.0
Counter throw	3.8	6.7				
Fall	3.8	6.7				
Not clear					7.4	
Ne-waza	11.3	33.3	0.0	26.5	14.8	31.3
Choke	3.8	6.7		8.8	3.7	
Armlock	5.7	13.3		10.3	3.7	
Not clear	1.9	13.3		7.4	7.4	
Other	3.8	13.3	28.0	0.0	0.0	18.8
Prohibited action	1.9	6.7	7.0			
Not clear	1.9	6.7	21.0			18.8

## Data Availability

The data supporting this systematic review are taken from previously reported studies and datasets, which have been cited. The processed data are available in the supplementary files.

## Additional Points

*What is already known?* (i) The injury incidence proportion in judo tournaments is wide ranging. (ii) Epidemiological data on judo injuries in tournaments can guide injury prevention programs and help researchers evaluate the effect of rule changes on the injury incidence. (iii) In the past two decades, judo competition rules have changed several times. Some of these rule changes were meant to reduce injury risk. *What are the new findings?* (i) The reported injury incidence proportions in judo are largely dependent on the injury definition and methodological approach. (ii) The head was the most common injury location overall, followed by the hand, knee, elbow, and shoulder. (iii) A sprain was the most common injury type, followed by contusions, lacerations, strains, and fractures.

## Supplementary Materials

Supplementary Materials
*Supplementary Appendix A.* Modified Appraisal Tool for Cross-Sectional Studies (AXIS). *Supplementary Appendix B.* The colour-coded table with the risk of bias assessments per question. *Supplementary Appendix C.* Distribution (in percentages %) between injured men and women during judo tournaments. *Supplementary Appendix D.* Injury incidence proportions for different age groups. *Supplementary Appendix E.* Distribution (in IR per 1000 AEs^*∗*^) of injuries across weight categories.
