# Supplementary material for: Epidemiology of Injuries during Judo Tournaments
Source: Transl Sports Med. 2023 Feb 18;2023:2713614. doi: 10.1155/2023/2713614 (PMC11022761; doi:10.1155/2023/2713614)
Supplement: Supplementary Materials — Supplementary Appendix A. Modified Appraisal Tool for Cross-Sectional Studies (AXIS). Supplementary Appendix B. The colour-coded table with the risk of bias assessments per question. Supplementary Appendix C. Distribution (in percentages %) between injured men and women during judo tournaments. Supplementary Appendix D. Injury incidence proportions for different age groups. Supplementary Appendix E. Distribution (in IR per 1000 AEs∗) of injuries across weight categories. [file 2713614.f1.zip › Supplementary Appendix A. v20220901.pdf]

Supplementary Appendix A

Modified Appraisal tool for Cross-Sectional Studies (AXIS)

	Question	Yes = 1 (or more)	No = 0	Not Applicable (Comment)
Introduction				
1	Were the aims/objectives of the study clear?			
Methods				
2	Was the study design appropriate for the stated aim(s)?			
3	Was the injury definition clearly stated?			
4	Was the exposure, i.e. number of matches, taken into account?			
5	Are the following outcomes described? 0.5 point per outcome: maximum total of 4.5 points<br>- injury incidence<br>- injury location<br>- Injury types (with discrimination between strain and sprain)<br>- Injury severity (including hospital treatment)<br>- Time-loss after injury<br>- Injury mechanism<br>- distribution of injuries across age-groups<br>- distribution of injuries across gender<br>- distribution of injuries across weight categories			
6	Were measures undertaken to address and categorise non-responders?			
7	Were the risk factor and outcome variables measured appropriate to the aims of the study?			

	Question	Yes = 1 (or more)	No = 0	Not Applicable (Comment)
8	Were the risk factor and outcome variables measured correctly using instruments/measurements that had been trialled, piloted or published previously?			
9	Is it clear what was used to determined statistical significance and/or precision estimates? (e.g. p-values, confidence intervals)			
10	Were the methods (including statistical methods) sufficiently described to enable them to be repeated?			
11	Was injury data collected prospectively? (Score = 2)<br>OR was injury data recalled retrospectively? (Score = 1)			
Results				
12	Were the basic data adequately described (including age, gender, weight and competition level of the participants)?			
13	Are there no concerns about non-response bias?			
14	If appropriate, was information about non-responders described?			
15	Were the results presented for all the analyses described in the methods?			
Discussion				
16	Were the authors' discussions and conclusions justified by the results?			
17	Were the limitations of the study discussed?			
Other				
18	Was there an absence of any funding sources or conflicts of interest that may affect the authors’ interpretation of the results?			
19	Was ethical approval or consent of participants obtained?			
	**Score =**<br><br>**Percentage =**			
