# Supplementary material for: Epidemiology of Injuries during Judo Tournaments
Source: Transl Sports Med. 2023 Feb 18;2023:2713614. doi: 10.1155/2023/2713614 (PMC11022761; doi:10.1155/2023/2713614)
Supplement: Supplementary Materials — Supplementary Appendix A. Modified Appraisal Tool for Cross-Sectional Studies (AXIS). Supplementary Appendix B. The colour-coded table with the risk of bias assessments per question. Supplementary Appendix C. Distribution (in percentages %) between injured men and women during judo tournaments. Supplementary Appendix D. Injury incidence proportions for different age groups. Supplementary Appendix E. Distribution (in IR per 1000 AEs∗) of injuries across weight categories. [file 2713614.f1.zip › Supplementary Appendix B. v20221229.pdf]

Supplementary Appendix B

Colour-code table with the risk of bias assessments per question

	Q1	Q2	Q3	Q4	Q5	Q6	Q7	Q8	Q9	Q10	Q11	Q12	Q13	Q14	Q15	Q16	Q17	Q18	Q19
**Beijsterveldt (2015)**																			
**Blach and Malliaropoulos (2021)**																			
**Blach and Smolders (2021)**																			
**Chirazi (2017)**																			
**Cierna (2019)**																			
**Dah (1989)**																			
**Didace (2017)**																			
**Engebretsen (2013)**																			
**Frey (2019)**																			
**Green (2007)**																			
**Ikumi (2019)**																			
**James (2003)**																			
**Junge (2009)**																			
**Laskowski (1995)**																			
**Lystad (2020)**																			
**Machado (2019)**																			
**Maciejewski and Callanta (2016)**																			
**Maciejewski and Pietkiewicz (2016)**																			
**Miarka (2018)**																			
**Park (2021)**																			
**Phillips (2001)**																			
**Pierantozzi (2009)**																			
**Pieter (2001)**																			
**Rousseau (2017)**																			
**Soligard (2017)**																			
