# Supplementary material for: Epidemiology of Injuries during Judo Tournaments
Source: Transl Sports Med. 2023 Feb 18;2023:2713614. doi: 10.1155/2023/2713614 (PMC11022761; doi:10.1155/2023/2713614)
Supplement: Supplementary Materials — Supplementary Appendix A. Modified Appraisal Tool for Cross-Sectional Studies (AXIS). Supplementary Appendix B. The colour-coded table with the risk of bias assessments per question. Supplementary Appendix C. Distribution (in percentages %) between injured men and women during judo tournaments. Supplementary Appendix D. Injury incidence proportions for different age groups. Supplementary Appendix E. Distribution (in IR per 1000 AEs∗) of injuries across weight categories. [file 2713614.f1.zip › Supplementary Appendix C. v20221229.pdf]

Supplementary Appendix C

Distribution (in percentages %) between injured men and women during judo tournaments.

Study	Gender	
	Men	Women
Cierna et al. (2019)	7.4%	9.2%
Blach and Malliaropoulos (2021)	5.0%	6.0%
Blach and Smolders (2021)*	54.9%	45.1%
Didace et al. (2017)*	*38.2%*	*72.7%*
Frey et al. (2019)	1.04%	1.33%
Green et al. (2007)	14.1%	12.0%
Ikumi et al. (2019)**	*21.1 (minor)*<br>*4.8 (major)*	*11.9 (minor)*<br>*4.9 (major)*
James et al. (2003)	14.3%	10.9%
Maciejewski and Pietkiewicz (2016)	10.7%	7.5%
Miarka et al. (2018)	11.1%	9.3%
Pieter et al. (2001)	7.0%	10.7%
Rousseau et al. (2017)	1.8%	2.6%

*Didace et al. (2017) and Blach and Smolders (2021) used the total number of injured judokas and expressed the proportion of men versus women.

**Ikumi et al. (2019) did not provide the exact numbers of the participating judokas. The shown numbers are the injury incidence rates per 1000 AEs. Severity of injury in this study was divided into two groups based on whether the injured judoka can continue to bout (minor injury), or if the injured judoka cannot continue to bout (major injury).
