# Supplementary material for: Epidemiology of Injuries during Judo Tournaments
Source: Transl Sports Med. 2023 Feb 18;2023:2713614. doi: 10.1155/2023/2713614 (PMC11022761; doi:10.1155/2023/2713614)
Supplement: Supplementary Materials — Supplementary Appendix A. Modified Appraisal Tool for Cross-Sectional Studies (AXIS). Supplementary Appendix B. The colour-coded table with the risk of bias assessments per question. Supplementary Appendix C. Distribution (in percentages %) between injured men and women during judo tournaments. Supplementary Appendix D. Injury incidence proportions for different age groups. Supplementary Appendix E. Distribution (in IR per 1000 AEs∗) of injuries across weight categories. [file 2713614.f1.zip › Supplementary Appendix D. v20221229.pdf]

Supplementary Appendix D

Injury incidence proportion for the different age groups

Study	Age	
	Youth (<18 years)	Adults (>18 years)
Frey et al. (2019)	0.9%	1.3%
Ikumi et al. (2019)	*53.6%**	*46.4%**
Machado et al. (2019)	14.4%	21.0%
Maciejewski and Pietkiewicz (2016)	4.7%	15.2%

*Ikumi et al. (2019) did not provide the exact numbers of the participating judokas. *The shown percentages are the distribution of injuries over youth and adults.*
