# Supplementary material for: Epidemiology of Injuries during Judo Tournaments
Source: Transl Sports Med. 2023 Feb 18;2023:2713614. doi: 10.1155/2023/2713614 (PMC11022761; doi:10.1155/2023/2713614)
Supplement: Supplementary Materials — Supplementary Appendix A. Modified Appraisal Tool for Cross-Sectional Studies (AXIS). Supplementary Appendix B. The colour-coded table with the risk of bias assessments per question. Supplementary Appendix C. Distribution (in percentages %) between injured men and women during judo tournaments. Supplementary Appendix D. Injury incidence proportions for different age groups. Supplementary Appendix E. Distribution (in IR per 1000 AEs∗) of injuries across weight categories. [file 2713614.f1.zip › Supplementary Appendix E. v20220901.pdf]

Supplementary Appendix E

Distribution (in IR per 1000 AEs*) of injuries across weight categories.

Weight category	Study				
	**Green et al. (2007)****	**Ikumi et al. (2019)*****		**Miarka et al. (2018)******	**Rousseau et al. (2017)*******
Men					
Lightweight	47	36.2		103.9	*1.3%*
Middleweight	32	38.4			*1.9%*
Heavyweight	39	21.2		126.9	*2.1%*
Women					
Lightweight	40	36.2		99.4	*2.2%*
Middleweight	36	38.4			*2.5%*
Heavyweight	50	21.2		70.6	*3.1%*

*Injury rate (IR) per 1000 athlete-exposures (AEs)

**Green et al. (2007) distributed 392 participants in the weight categories as follows: lightweight M<73 W<57, middleweight M<90 W<70 and heavyweight M+100 W+78.

***Ikumi et al. (2019) did not provide the distribution between men and women, and distributed the weight categories as follows: lightweight M<66 W<52, middleweight M<90 W<70 and heavyweight M+100 W+78.

****Miarka et al. (2018) distributed 673 participants in the weight categories as follows: lightweight M<81 W<63 and heavyweight M+100 W+78.

*****Rousseau et al (2017) did not provide the injury rate per 1000 athlete-exposures, and distributed 2458 participants in the weight categories as follows: lightweight M<66 W<52, middleweight M<90 W<70 and heavyweight M+100 W+78.
